# Supplementary material for: Effect of Different Enological Tannins on Oxygen Consumption, Phenolic Compounds, Color and Astringency Evolution of Aglianico Wine
Source: Molecules. 2020 Oct 10;25(20):4607. doi: 10.3390/molecules25204607 (PMC7587184; doi:10.3390/molecules25204607)
Supplement: Supplementary file 1 [file molecules-25-04607-s001.pdf]

Effect of Different Enological Tannins on Oxygen Consumption, Phenolic Compounds, Color and Astringency Evolution of Aglianico Wine

Luigi Picariello [1], Alessandra Rinaldi [1,2], Martino Forino [1], Francesco Errichiello [1], Luigi Moio [1] and Angelita Gambuti [1,*]

[1] Department of Agricultural Sciences, Grape and Wine Science Division, University of Naples "Federico II", Viale Italia (Angolo Via Perrottelli), 83100 Avellino, Italy; luigi.picariello@unina.it (L.P.); alessandra.rinaldi@unina.it (A.R.); forino@unina.it (M.F.); francescoerrichiellojr@gmail.com (F.E.); luigi.moio@unina.it (L.M.)

[2] Biolaffort, 126 Quai de la Souys, 33100 Bordeaux, France

* Correspondence: angelita.gambuti@unina.it; Tel.: +39-081-2532605:

Figure S1: ^{1}H-NMR spectrum in CD_3OD of the TT mixture. Resonances marked with red asterisks are relative to Epigallocatechin-3-*O*-gallate and resonances marked with green asterisks are relative to Epigallocatechin

Figure S2: Enlargements of the HR-ESIMS spectrum of the TT mixture acquired in the negative ione mode

Figure S3: ^{1}H-NMR spectrum in CD_3OD of the CT mixture

Figure S4: Enlargements of the HR-ESIMS spectrum of the CT mixture acquired in the negative ione mode

Figure S5: ^{1}H-NMR spectrum in CD_3OD of the ET mixture

Figure S6: Enlargements of the HR-ESIMS spectrum of the ET mixture acquired in the negative ione mode

Figure S7: ^{1}H-NMR spectrum in CD_3OD of the GT mixture

Figure S8: HR-ESIMS spectrum of the GT mixture acquired in the negative ione mode

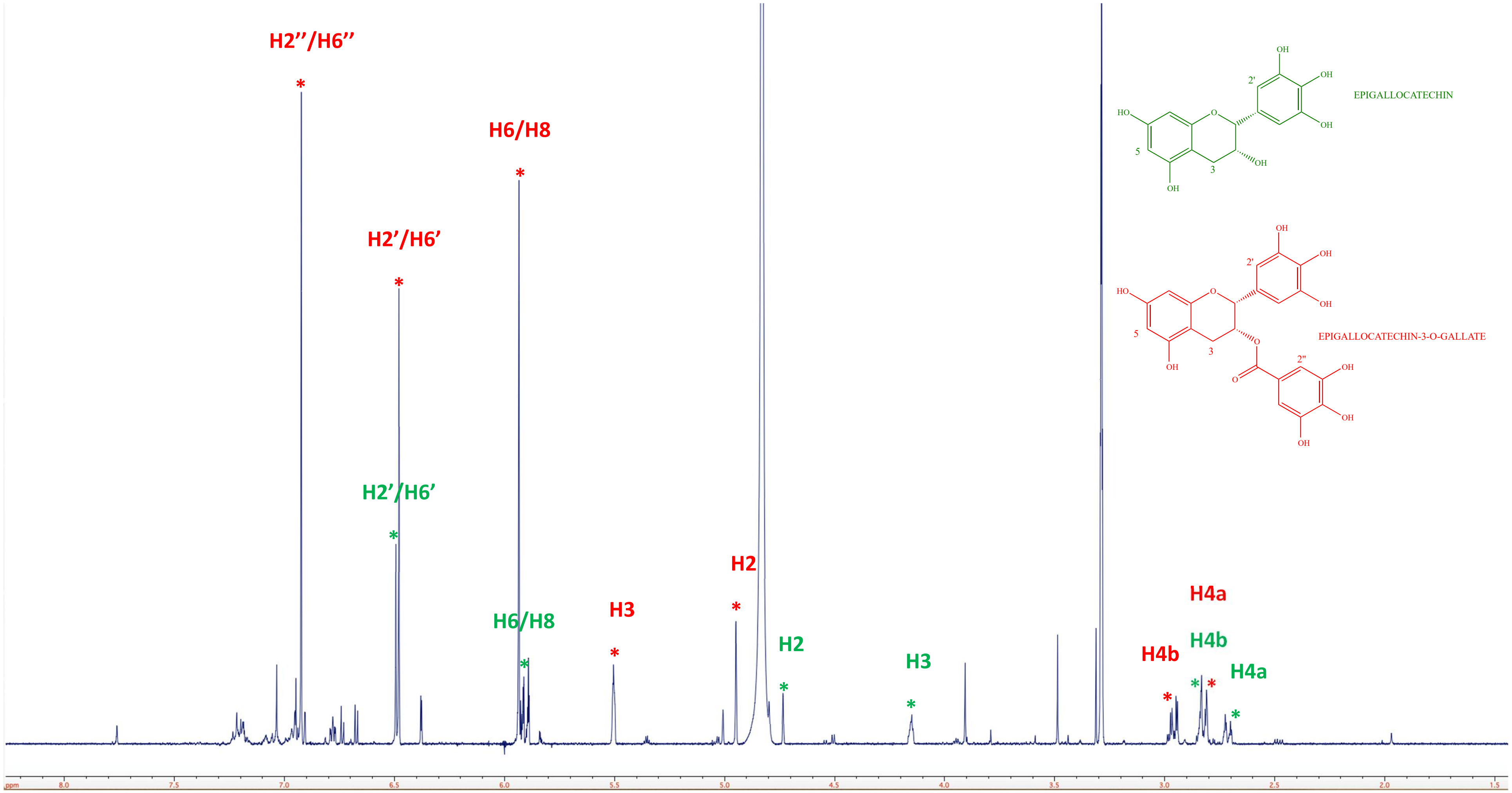

Figure 1 in supplementary material: ^{1}H-NMR spectrum in CD_3OD of the TT mixture. Resonances marked with red asterisks are relative to Epigallocatechin-3-*O*-gallate and resonances marked with green asterisks are relative to Epigallocatechin.

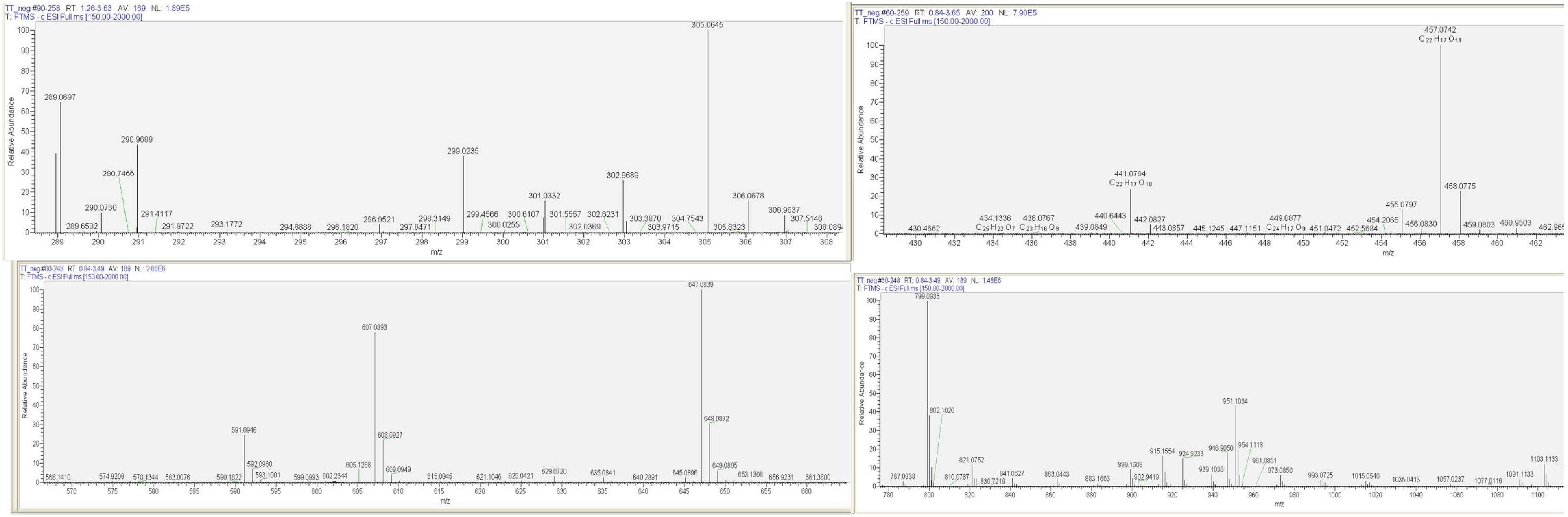

Figure 2 in supplementary material: Enlargements of the HR-ESIMS spectrum of the TT mixture acquired in the negative ione mode

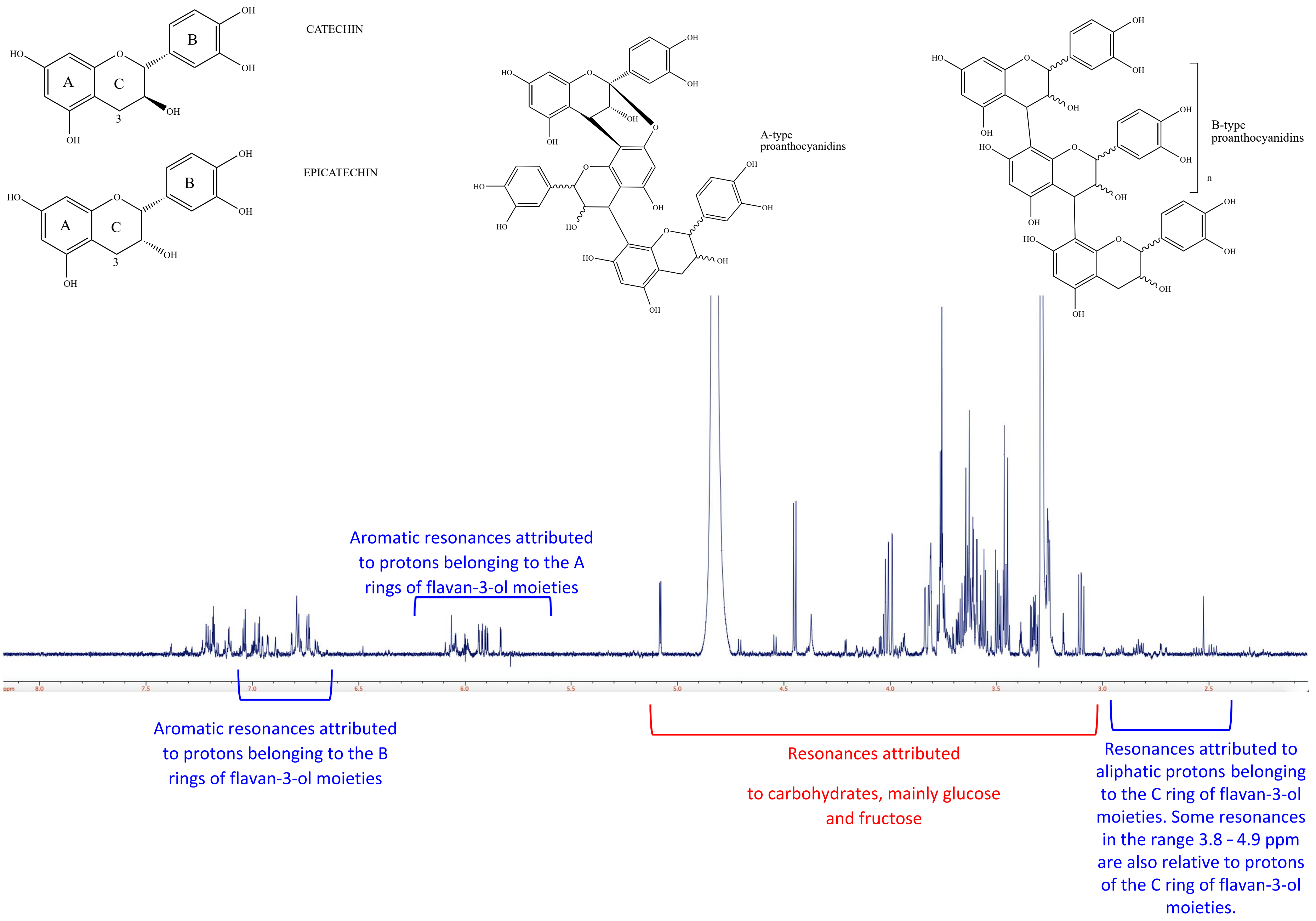
CATECHIN
EPICATECHIN
A
B
C
3
HO
OH
O
A-type proanthocyanidins
B-type proanthocyanidins
n
ppm
8.0
7.5
7.0
6.5
6.0
5.5
5.0
4.5
4.0
3.5
3.0
2.5
Aromatic resonances attributed to protons belonging to the A rings of flavan-3-ol moieties
Aromatic resonances attributed to protons belonging to the B rings of flavan-3-ol moieties
Resonances attributed
to carbohydrates, mainly glucose and fructose
Resonances attributed to aliphatic protons belonging to the C ring of flavan-3-ol moieties. Some resonances in the range 3.8 – 4.9 ppm are also relative to protons of the C ring of flavan-3-ol moieties.

Figure 3 in supplementary material: ^{1}H-NMR spectrum in CD_3OD of the CT mixture

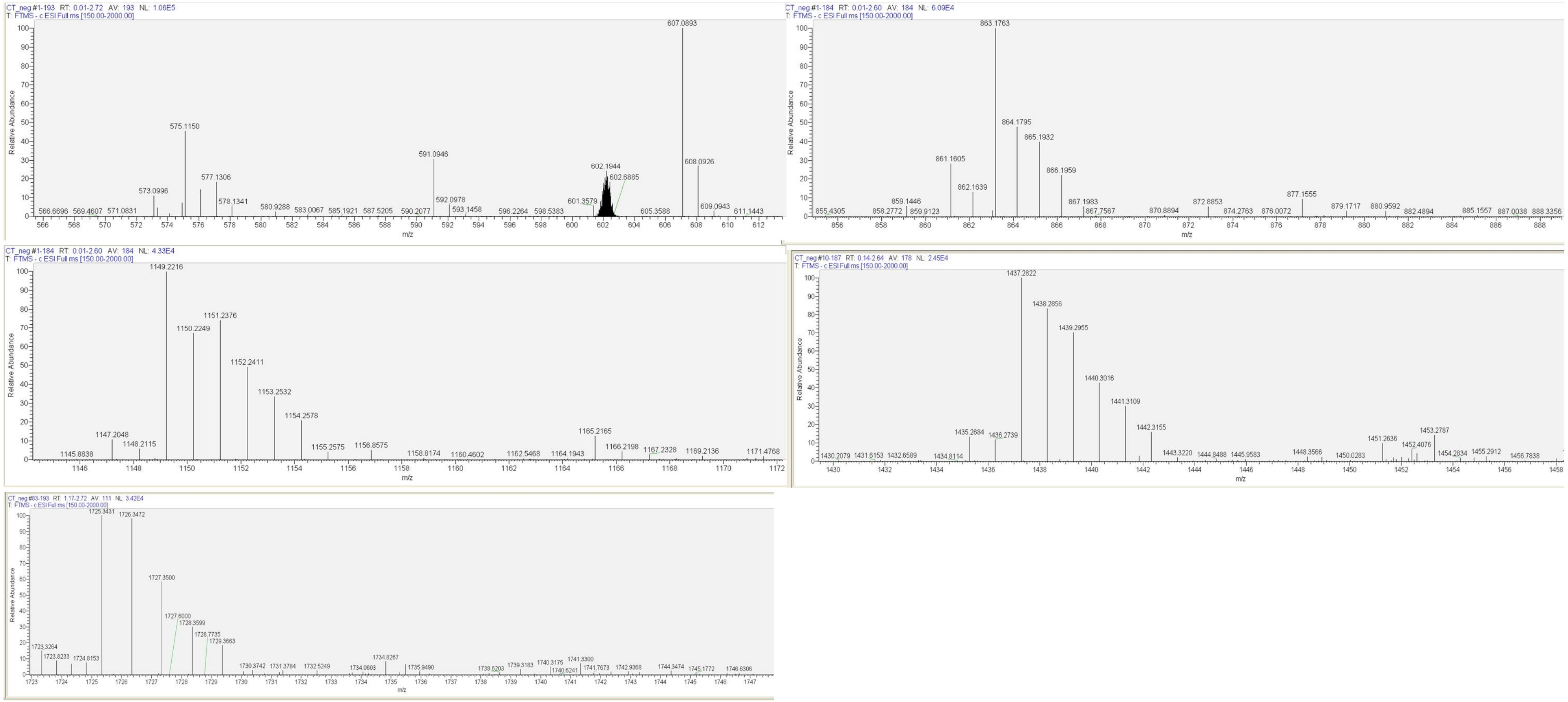

Figure 4 in supplementary material: Enlargements of the HR-ESIMS spectrum of the CT mixture acquired in the negative ione mode

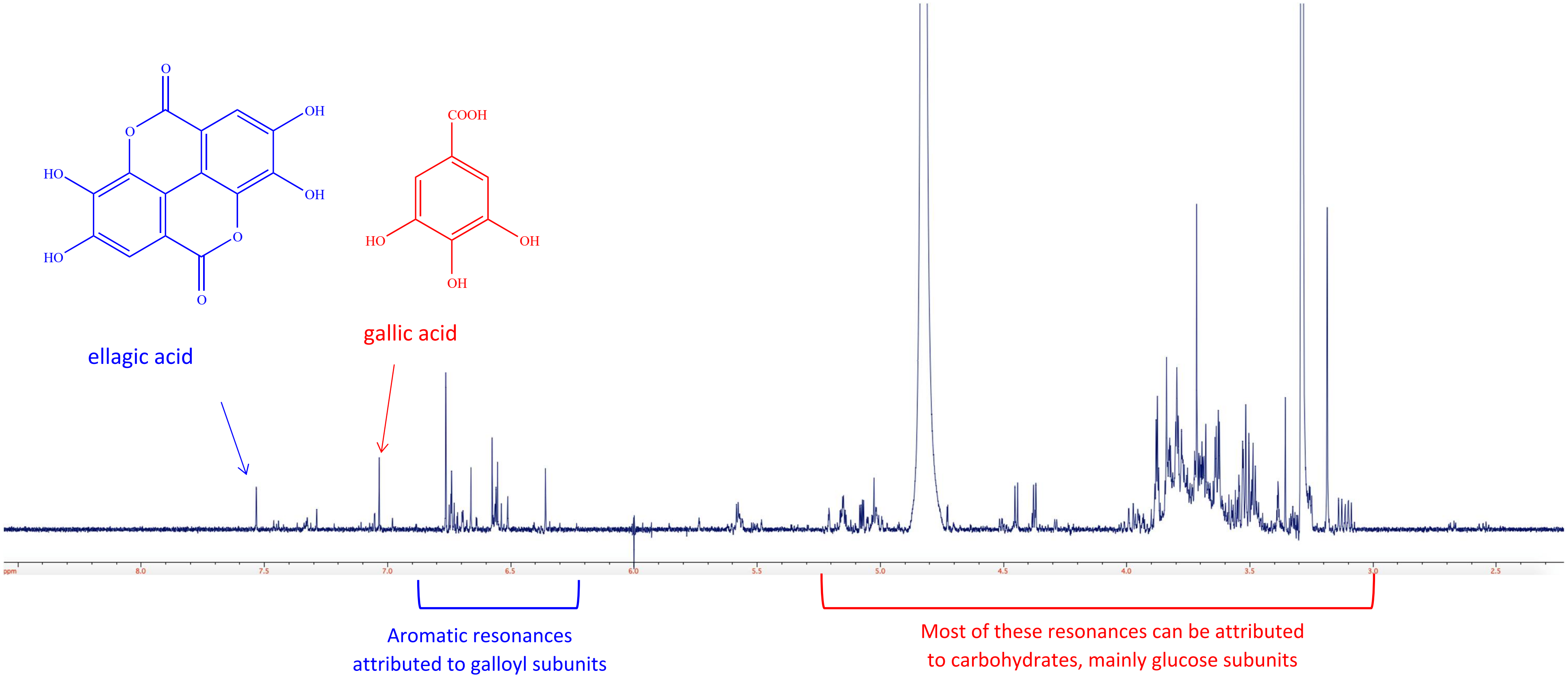

Figure 5 in supplementary material: ^{1}H-NMR spectrum in CD_3OD of the ET mixture.

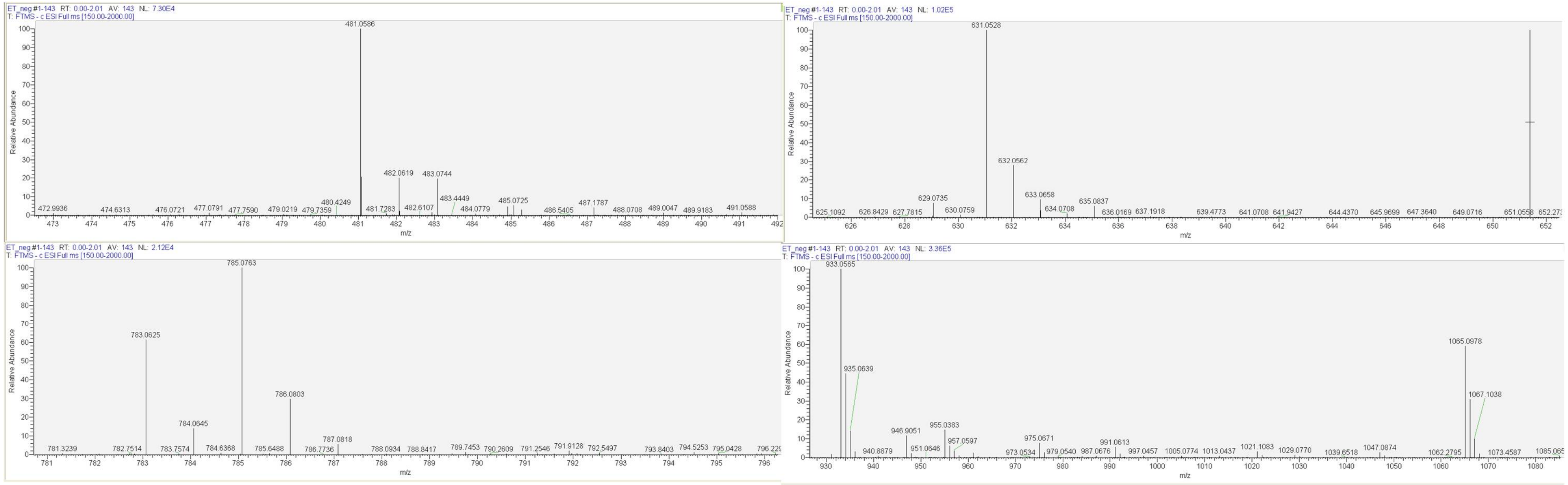

Figure 6 in supplementary material: Enlargements of the HR-ESIMS spectrum of the ET mixture acquired in the negative ione mode

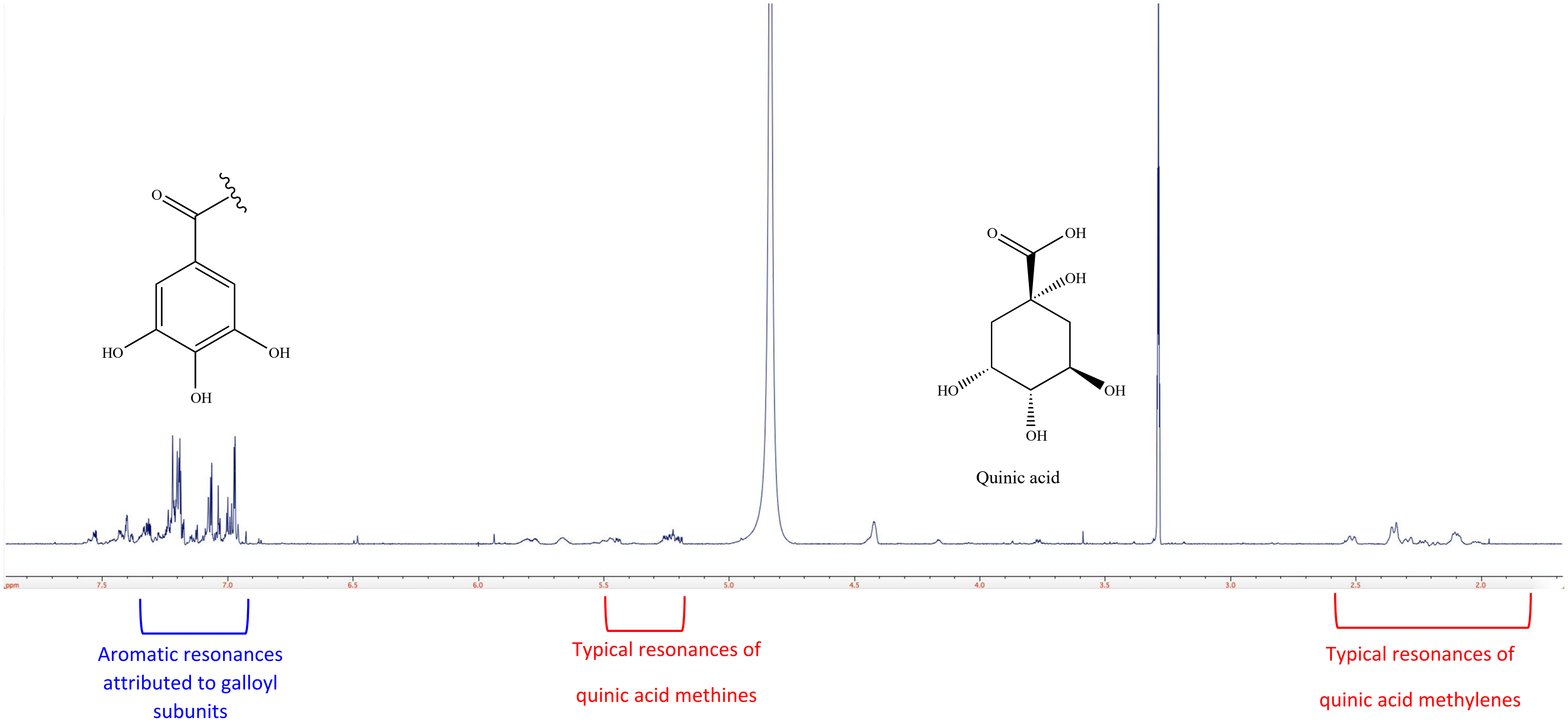

Figure 7 in supplementary material: ^{1}H-NMR spectrum in CD_3OD of the GT mixture.

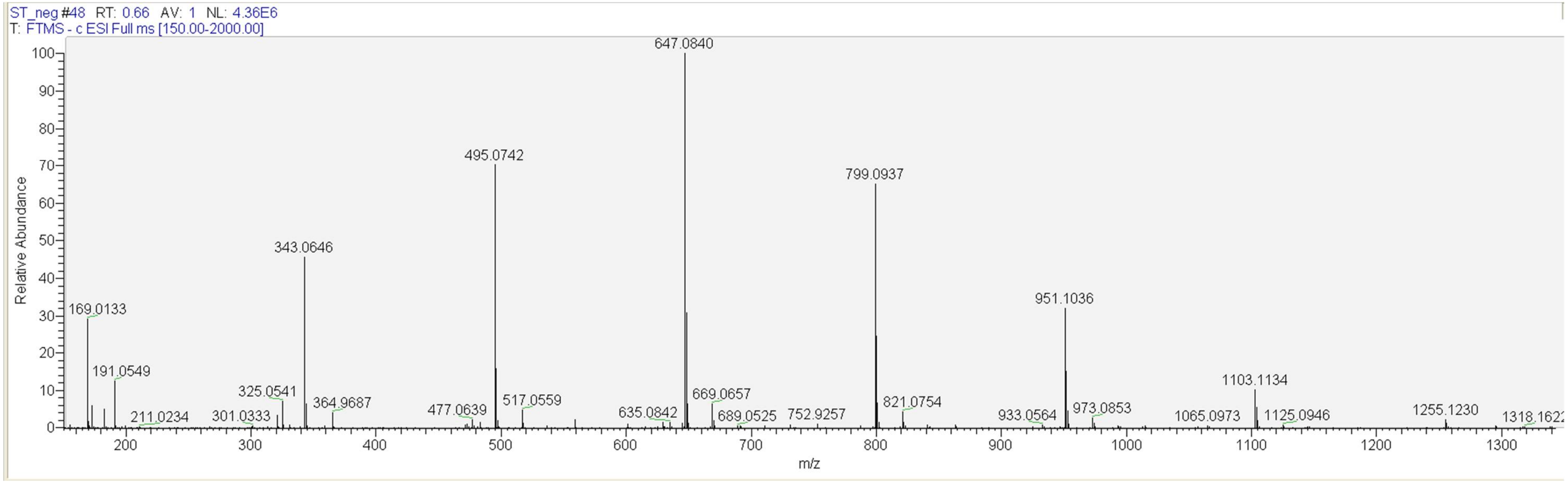

Figure 8 in supplementary material: HR-ESIMS spectrum of the GT mixture acquired in the negative ione mode
